# EHMTI-0334. Chronic migraine in young age: clinical characteristics in a prospective chronic migraine registry

**DOI:** 10.1186/1129-2377-15-S1-D24

**Published:** 2014-09-18

**Authors:** M Ruiz, MI Pedraza, C de la Cruz, C Rodríguez, MS Hernandez, M de Lera, I Muñoz, R Moreno, J Barón, A Guerrero

**Affiliations:** 1 Neurologist, Hospital Clínico Universitario de Valladolid, Valladolid, Spain

## Introduction

Chronic migraine (CM) prevalence peaked in midlife, but it is also present among young age.

## Aim

We aimed to analyze characteristics of young patients in a CM prospective registry.

## Methods

Patients firstly attended in an outpatient headache office in a tertiary hospital (January 2013-May 2014). CM diagnosed accordingly to ICHD-2R criteria. We gathered demographic and clinical data including comorbidities and risk factors. Patients were classified in two groups, from 14 to 25 years old (Group A), and older than 25 (Group B).

## Results

218 patients (34 males, 184 females), mean age of 40.2 ± 14.2 years (14-71) were diagnosed of CM. 36 cases (16.5%, 8 male, 28 female) were included in Group A. Among young patients, considering risk factors, in 15 (41.7%) stressful life events, in 2 (5.6%) mood disorders, and in 3 (8.4%) obesity. Among comorbidities, in 17 cases (47.2 %) vascular risk factor, especially smoking, in 2 (5.6 %) respiratory disease and no case with other chronic pain. 5 of 28 female patients (17.8%) described menstrually-related migraine. When comparing both groups, age at onset of migraine (13.1 ± 4.8 vs 19.3 ± 9, p<0.001), time in months from onset of CM (12.1 ± 11.3 vs 48.2 ± 80.5, p<0.001), and percentage of patients with medication overuse (27.8% vs 78.6%, p<0.001) and previous preventive therapies (27.8% vs 51.1%, p:0.01) were significantly lower in Group A patients.

## Conclusion

CM is not uncommon among young age in our registry. Medication overuse is less present in young patients with CM

No conflict of interest.

